# Quake: quality-aware detection and correction of sequencing errors

**DOI:** 10.1186/gb-2010-11-11-r116

**Published:** 2010-11-29

**Authors:** David R Kelley, Michael C Schatz, Steven L Salzberg

**Affiliations:** 1 Center for Bioinformatics and Computational Biology, Institute for Advanced Computer Studies, and Department of Computer Science, University of Maryland, College Park, MD 20742, USA; 2 Simons Center for Quantitative Biology, Cold Spring Harbor Laboratory, 1 Bungtown Road, Cold Spring Harbor, NY 11724, USA

## Abstract

We introduce Quake, a program to detect and correct errors in DNA sequencing reads. Using a maximum likelihood approach incorporating quality values and nucleotide specific miscall rates, Quake achieves the highest accuracy on realistically simulated reads. We further demonstrate substantial improvements in *de novo *assembly and SNP detection after using Quake. Quake can be used for any size project, including more than one billion human reads, and is freely available as open source software from http://www.cbcb.umd.edu/software/quake.

## Rationale

Massively parallel DNA sequencing has become a prominent tool in biological research [1,2]. The high-throughput and low cost of second-generation sequencing technologies has allowed researchers to address an ever-larger set of biological and biomedical problems. For example, the 1000 Genomes Project is using sequencing to discover all common variations in the human genome [3]. The Genome 10K Project plans to sequence and assemble the genomes of 10,000 vertebrate species [4]. Sequencing is now being applied to a wide variety of tumor samples in an effort to identify mutations associated with cancer [5,6]. Common to all of these projects is the paramount need to accurately sequence the sample DNA.

DNA sequence reads from Illumina sequencers, one of the most successful of the second-generation technologies, range from 35 to 125 bp in length. Although sequence fidelity is high, the primary errors are substitution errors, at rates of 0.5-2.5% (as we show in our experiments), with errors rising in frequency at the 3' ends of reads. Sequencing errors complicate analysis, which normally requires that reads be aligned to each other (for genome assembly) or to a reference genome (for detection of mutations). Mistakes during the overlap computation in genome assembly are costly: missed overlaps may leave gaps in the assembly, while false overlaps may create ambiguous paths or improperly connect remote regions of the genome [7]. In genome re-sequencing projects, reads are aligned to a reference genome, usually allowing for a fixed number of mismatches due to either SNPs or sequencing errors [8]. In most cases, the reference genome and the genome being newly sequenced will differ, sometimes substantially. Variable regions are more difficult to align because mismatches from both polymorphisms and sequencing errors occur, but if errors can be eliminated, more reads will align and the sensitivity for variant detection will improve.

Fortunately, the low cost of second-generation sequencing makes it possible to obtain highly redundant coverage of a genome, which can be used to correct sequencing errors in the reads before assembly or alignment. Various methods have been proposed to use this redundancy for error correction; for example, the EULER assembler [9] counts the number of appearances of each oligonucleotide of size *k *(hereafter referred to as *k*-mers) in the reads. For sufficiently large *k*, almost all single-base errors alter *k*-mers overlapping the error to versions that do not exist in the genome. Therefore, *k*-mers with low coverage, particularly those occurring just once or twice, usually represent sequencing errors. For the purpose of our discussion, we will refer to high coverage *k*-mers as *trusted*, because they are highly likely to occur in the genome, and low coverage *k*-mers as *untrusted*. Based on this principle, we can identify reads containing untrusted *k*-mers and either correct them so that all *k*-mers are trusted or simply discard them. The latest instance of EULER determines a coverage cutoff to separate low and high coverage *k*-mers using a mixture model of Poisson (low) and Gaussian (high) distributions, and corrects reads with low coverage *k*-mers by making nucleotide edits to the read that reduce the number of low coverage *k*-mers until all *k*-mers in the read have high coverage [10]. A number of related methods have been proposed to perform this error correction step, all guided by the goal of finding the minimum number of single base edits (edit distance) to the read that make all *k*-mers trusted [11-14].

In addition, a few alternative approaches to error correction should be mentioned. Past methods intended for Sanger sequencing involve multiple sequence alignments of reads rendering them infeasible for short read datasets [15-17]. More recently, a generalized suffix tree of the reads was shown to be an effective data structure for detecting and correcting errors in short reads [18,19]. De Bruijn graph-based short read assemblers [10,11,13,20,21] perform substantial error correction of reads in the de Bruijn graph. For example, short dead end paths are indicative of a sequencing error at the end of a read and can be removed, and 'bubbles' where a low coverage path briefly diverges from and then reconnects to high coverage nodes are indicative of sequencing errors at the middle of a read and can be merged. Finally, a number of methods have been proposed to cluster reads and implicitly correct sequencing errors in data where the targets vary in abundance such as sequencing of small RNAs or 16 s rRNA [22-25].

Although methods that search for the correct read based on minimizing edit distance will mostly make the proper corrections, edit distance is an incomplete measure of relatedness. First, each position in a sequencing read is assigned a quality value, which defines the probability that the basecall represents the true base. Though questions have been raised about the degree to which quality values exactly define the probability of error [26], newer methods for assigning them to base calls demonstrate substantial improvements [27-31], and for our purpose of error correction, the quality values can be useful even if they only rank one base as more likely to be an error as another. We should prefer to edit a read at these lower quality bases where errors are more likely, but edit distance treats all bases the same regardless of quality. Furthermore, specifics of the Illumina technology cause certain miscalls to be more likely than others. For example, bases are called by analysis of fluorescent output from base-incorporating chemical reactions, and A and C share a red detection laser while G and T share a green detection laser. Thus, A and C are more likely to be mistaken for each other than for G or T [26]. Edit distance treats all error substitutions as equally likely.

In this paper, we introduce a new algorithm called Quake to correct substitution errors in sets of DNA sequencing reads produced as part of >15× coverage sequencing projects, which has become commonplace thanks to the efficiency of second-generation sequencing technologies. Quake uses the *k*-mer coverage framework, but incorporates quality values and rates of specific miscalls computed from each sequencing project. In addition, Quake incorporates a new method to choose an appropriate coverage cutoff between trusted *k*-mers (those that are truly part of the genome) and erroneous *k*-mers based on weighting *k*-mer counts in the reads using the quality values assigned to each base. On simulated data using quality values from real reads, Quake is more accurate than previous methods, especially with relatively long Illumina reads. Correcting reads guided by edit distance alone, without the use of quality values, results in many more improperly corrected reads. These reads are then chimeric, containing sequence from two distinct areas of the genome, which can be a major problem for assembly software.

Finally, we explore the impact of error correction with Quake on two important bioinformatics applications - *de novo *assembly and detection of variations with respect to a reference genome. Even a sophisticated assembler such as Velvet [20], which performs its own error correction using the assembly graph, benefits from pre-processing the reads with Quake. SOAPdenovo [13], a parallel assembler capable of assembling mammalian-size datasets, also produces better assemblies after error correction. For variant detection, correcting errors before mapping reads to a reference genome results in more reads aligned to SNP locations and more SNPs discovered. Note that Quake and other correction methods that rely on coverage of *k*-mers are inappropriate for applications where low coverage does not necessary implicate a sequencing error such as metagenomics, RNA-Seq, and ChIP-Seq.

Quake is freely available as open source software from our website [32] under the Perl Artistic License [33].

## Results and discussion

### Accuracy

The two goals of error correction are to cleanly separate reads with errors from reads without errors and to properly correct the reads with errors. To assess Quake's ability to accurately complete these tasks, we simulated sequencing reads with errors from finished genomes (using an approach comparable to the 'Maq simulate' program [34]) and compared Quake's corrections to the true reference. For each dataset, we categorized reads and their corrections into four outcomes. As positive outcomes, we counted the number of reads that were properly corrected to their original state or trimmed such that no errors remained. As negative outcomes, we counted the number of reads mis-corrected producing a false sequence or left uncorrected even though they contained errors. Reads were simulated by choosing a position in the reference genome, using the quality values from an actual Illumina sequencing read, and changing the nucleotides according to the probabilities defined by those quality values. Dohm *et al*. measured the bias in Illumina specific nucleotide to nucleotide miscall rates by sequencing reads from *Helicobacter acinonychis *and *Beta vulgaris*, aligning them to high quality reference genomes, and counting the number of each type of mismatch in the alignments [26]. At simulated errors, we changed the nucleotide according to these frequencies.

To compare Quake's accuracy to that of previous error correction programs, we corrected the reads using EULER [10], Shrec [18], and SOAPdenovo [13] on a four core 2.4 GHz AMD Opteron machine. Quake and the other *k*-mer based correction tools used *k *= 15. SOAPdenovo's error correction module does not contain a method to choose the cutoff between trusted and untrusted *k*-mers, so we tried a few appropriate values and report the best results. We similarly tried multiple values for Shrec's strictness parameter that is used to help differentiate true and error reads via coverage. These are very sensitive parameters, and leaving them to the user is a critical limitation of these programs. Alternatively, EULER and Quake determine their parameters automatically using the data.

Table 1 displays the average of the accuracy statistics after five iterations of simulated 36 bp reads to 40× coverage (5.5 M reads) from *E. coli *536 [GenBank: NC_008253]. Quality value templates were taken from the sequencing of *E. coli *K12 substrain MG1655 [SRA:SRX000429]. The datasets contained an average of 1.17 M reads with errors. Of the reads that Quake tried to correct, 99.83% were corrected accurately to the true sequence. Quake properly corrected 88.3% (90.5% including trims) of error reads, which was 6.9% more reads than the second best program SOAPdenovo, made 2.3× fewer mis-corrections than SOAPdenovo, and allowed 1.8× fewer reads with errors. The 5265.4 error reads that Quake keeps have errors that only affect a few *k*-mers (at the end of the read), and these *k*-mers happen to exist elsewhere in the genome. We could not successfully run EULER on these short reads.

**Table 1:** Simulated 36 bp E. coli

	Corrections	Trim corrections	Mis-corrections	Error reads kept	Time (min)
Quake	1035709.4	26337.0	1744.0	5537.0	14.2
SOAPdenovo	969666.4	120529.0	3912.8	9288.4	12.4
Shrec	964431.8	0.0	165422.0	41733.6	87.6

Simulated *E. coli *36 bp reads at 40× coverage averaged over five runs. For each method, we counted the number of reads that were properly corrected to their original state (Corrections), trimmed such that no errors remained (Trim corrections), mis-corrected to false sequence (Mis-corrections), and contained errors but were kept in the set (Error reads kept). Quake corrects more reads while mis-correcting fewer reads and keeping fewer reads with errors than all programs.

We performed the same test using five iterations on 40× coverage (1.6 M reads) of 124 bp reads from *E. coli 536*. Most of these reads had very low quality suffixes expected to contain many errors. Quake handled these reads seamlessly, but the other programs produced very poor results. Thus, we first trimmed every read *r *to the length

(1) $$l=\underset{x}{\arg\;\max}\sum_{i=x}^{\left|r\right|}t-q_{i}$$

By setting *t *= 3, we mainly trim nucleotides with quality value 2 off the ends of the reads, but will trim past a higher quality base call if there are a sufficient number of nucleotides with quality ≤2 preceding it. On this data (where full results are displayed in Table 2), Quake is 99.9% accurate on reads that it tries to correct. Of the 297 K error reads, Quake corrected 95.6% (97.9% including trims), 2.5% more than SOAPdenovo, the second most effective program. However, SOAPdenovo makes many more mistakes on the longer reads by mis-correcting 28.9× more reads and keeping 11.9× more reads with errors in the set. Shrec and EULER correct far fewer reads and mis-correct more reads than Quake.

**Table 2:** Simulated 124 bp E. coli

	Corrections	Trim corrections	Mis-corrections	Error reads kept	Time (min)
Quake	283769.4	6581.2	243.0	393.6	11.8
SOAPdenovo	276770.4	2942.6	7019.4	5490.2	16.9
Shrec	165942.7	0.0	33140.3	96626.7	97.1
EULER	228316.4	16577.4	3763.0	414.8	6.9

Simulated *E. coli *124 bp reads at 40× coverage averaged over five runs. Column descriptions are the same as Table 1. Quake corrects more reads while mis-correcting far fewer reads and keeping fewer reads with errors than all programs.

To demonstrate Quake's ability to scale to larger genomes, we simulated 325 million 124 bp reads from the 249 Mbp human chromosome 1 (version hg19), which provided 34× coverage after trimming. Due to the larger size of the sequencing target, we counted and corrected 18-mers in the reads. Of the 15.23 M reads containing errors, Quake corrected 12.83 M (84.2%) and trimmed to a correct prefix another 0.82 M (5.4%). Because we could not successfully run SOAPdenovo using 18-mers, we corrected using 17-mers, a reasonable choice given that the authors of that software chose to correct reads using 17-mers for the entire human genome [13]. Quake corrected 11% more reads than SOAPdenovo, reduced mis-corrections by 64%, and kept 15% fewer error reads. EULER produced very poor correction results, for example, correcting less than half as many reads as Quake with more mis-corrections and error reads kept. On a dataset this large, Shrec required more memory than our largest computer (256 GB).

Relative to the 124 bp simulated reads from *E. coli*, Quake's attempted corrections were accurate at a lower rate (99.02%) and Quake kept more error reads in the dataset (1.11 M, 7.27%). This is caused by the fact that the human genome contains far more repetitive elements than *E. coli*, such as the LINE and SINE retrotransposon families [35]. The more repetitive the genome is, the greater the chance is that a sequencing error will merely change one trusted *k*-mer to another trusted *k*-mer, hiding the error. To quantify this property of the two genomes, we computed the percentage of all possible single base mutations to *k*-mers in each genome which create *k*-mers that also exist in the genome. In *E. coli 536*, this is true for 2.25% of 15-mer mutations, and in chromosome 1 of the human genome, it is true for 13.8% of 18-mer mutations. Increasing the *k*-mer size does little to alleviate the problem as still 11.1% of 19-mer mutations are problematic. Nevertheless, allowing a small percentage of error reads may not be terribly problematic for most applications. For example, genome assemblers will notice the lower coverage on the paths created by these reads and clean them out of the assembly graph.

### Genome assembly

**Figure 1:**
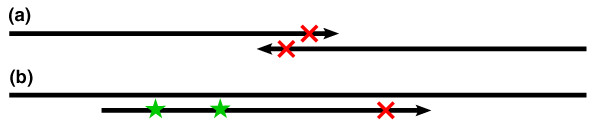
**Alignment difficulty**. Detecting alignments of short reads is more difficult in the presence of sequencing errors (represented as X's). (a) In the case of genome assembly, we may miss short overlaps between reads containing sequencing errors, particularly because the errors tend to occur at the ends of the reads. (b) To find variations between the sequenced genome and a reference genome, we typically first map the reads to the reference. However, reads containing variants (represented as stars) and sequencing errors will have too many mismatches and not align to their true genomic location.

In *de novo *genome assembly, the goal is to build contiguous and unambiguous sequences called contigs from overlapping reads. The traditional formulation of the assembly problem involves first finding all overlaps between reads [36], taking care to find all true overlaps between reads sequenced from the same genome location and avoid false overlaps between reads sequenced from remote regions [7]. Because of sequencing errors, we must allow mismatches in the overlap alignments to find all true overlaps, but we cannot allow too many or false overlaps will be found and fragment the assembly. With short reads, we must allow a short minimum overlap length, but in the presence of sequencing errors, particularly when these errors tend to occur at the ends of the reads, we may frequently overlook true overlaps (see Figure 1). A de Bruijn graph formulation of the assembly problem has become very popular for short reads [10,11,13,20], but is very sensitive to sequencing errors. A substantial portion of the work performed by these programs goes towards recognizing and correcting errors in the graph.

Having established the accuracy of Quake for error correction on simulated data, we measured the impact of Quake on genome assembly by assembling the reads before and after error correction. One assembly is better than another if it is more connected and more accurately represents the sequenced genome. To measure connectedness, we counted the number of contigs and scaffolds in the assembly larger than 50 bp as well as the N50 and N90 for each, which is the contig/scaffold size for which 50% (90%) of the genome is contained in contigs/scaffolds of equal or larger size. Fewer contigs/scaffolds and larger N50 and N90 values signify that the reads have been more effectively merged into large genomic sequences. In addition, we counted the number of reads included in the assembly because greater coverage generally leads to better accuracy in consensus calling. When a reference genome was available, we used it to validate the correctness of the assembly. We aligned all scaffolds to the reference using MUMmer [37] and considered scaffolds that did not align for their entire length (ignoring 35 bp on each end) at >95% identity to be mis-assembled. We also counted the number of single base differences between the reference and otherwise properly assembled scaffolds. Finally, we computed the percentage of reference nucleotides covered by some aligning scaffold.

Velvet is a widely used de Bruijn graph-based assembler that performs error correction by identifying graph motifs that signify sequencing errors [20], but does not use a stand-alone error correction module like EULER [10] or SOAPdenovo [13]. Thus, we hypothesized that Quake would help Velvet produce better assemblies. To test this hypothesis, we corrected and assembled 152× (20.8 M reads) coverage of 36 bp reads from *E. coli *K12 substrain MG1655 [SRA:SRX000429]. We used Velvet's option for automatic computation of expected coverage and chose the de Bruijn graph *k*-mer size that resulted in the best assembly based on the connectedness and correctness statistics discussed above.

Table 3 displays the assembly statistics for *E. coli *with Velvet. Quake corrected 2.44 M (11.7%) and removed 0.57 M (2.8%) reads from the dataset. After correction, 0.75 M (3.8%) more reads were included in the assembly, which contained 13% fewer contigs and 13% fewer scaffolds. Though this significant increase in connectedness of the assembly does not manifest in the N50 values, which are similar for both assemblies, the contig N90 increases by 47% and the scaffold N90 increases by 11%. With respect to correctness, the corrected read assembly contained one fewer mis-assembled scaffold and 31% fewer mis-called bases, and still covered slightly more of the reference genome. This improvement was consistent in experiments holding out reads for lesser coverage of the genome (data not shown). As the coverage decreases, the distributions of error and true *k*-mers blend together and the choice of cutoff must carefully balance making corrections and removing useful reads from low coverage regions. On this dataset, the minimum coverage at which the assembly improved after correction using Quake was 16×.

**Table 3:** Velvet E. coli assembly

	Contigs	N50	N90	Scaffolds	N50	N90	Breaks	Miscalls	Cov
Uncorrected	398	94,827	17,503	380	95,365	23,869	5	456	0.9990
Corrected	345	94,831	25,757	332	95,369	26,561	4	315	0.9992

Velvet assemblies of *E. coli *36 bp paired end reads at 152× coverage. After correcting the reads, more reads are included in the assembly into fewer contigs and scaffolds. N50 and N90 values were computed using the genome size 4,639,675 bp. The N50 value was similar for both assemblies, but N90 grew significantly with corrected reads. Correcting the reads also improved the correctness of the assembly producing fewer mis-assembled scaffolds (Breaks) and miscalled bases (Miscalls) and covering a greater percentage of the reference genome (Cov).

We also measured Quake's impact on a larger assembly with longer reads by assembling 353.7 M Illumina reads, all of them 124 bp in length, from the alfalfa leafcutting bee *Megachile rotundata*, with an estimated genome size of 300 Mbp. (Contact the corresponding author for details on data access.) Assembly was performed with SOAPdenovo [13] using a de Bruijn graph *k*-mer size of 31 and the '-R' option to resolve small repeats. Assembly of the raw uncorrected reads was quite poor because of the very low quality suffixes of many of the 124 bp reads. Thus, we compare assembly of quality trimmed reads (performed as described above), reads corrected using Quake, and trimmed reads corrected with SOAPdenovo's own error correction module. Quake and SOAPdenovo corrected using 18-mers and a coverage cutoff of 1.0.

Correcting errors in the reads had a significant affect on the quality of the assembly as seen in Table 4. In the Quake assembly, >123 K fewer contigs were returned as contig N50 grew by 71% and contig N90 more than doubled compared to the standard approach of only trimming the reads before assembly. Similarly to the simulated reads, Quake is able to correct more reads than SOAPdenovo, which leads to 1.5% more reads included in the assembly than SOAPdenovo and slightly more than the assembly of uncorrected reads. Improvements to the connectedness statistics compared to SOAPdenovo were modest. Surprisingly, although nearly 2.5× fewer scaffolds were returned after error correction with Quake, scaffold N50 remained virtually the same and N90 slightly decreased. We investigated a few possible explanations for this with inconclusive results; for example, scaffold sizes did not improve substantially after adding back mate pairs 8 excluded due to uncorrectable errors. Because N50 and N90 can be somewhat volatile and the scaffolds in the *E. coli *assembly above did improve after error correction, this is potentially an artifact of this particular dataset, that is the library sizes used with respect to the repeat structure of the genome.

**Table 4:** SOAPdenovo bee assembly

Assembly	Trimmed Only	Corrected	Removed	Contigs	N50	N90	Scaffolds	N50	N90	Reads
Uncorrected Corrected	146.0 M	-	12.9 M	312,414	2,383	198	90,201	37,138	9,960	167.3 M
SOAPdenovo Corrected	134.4 M	15.7 M	15.6 M	188,480	4,051	515	36,525	36,525	9,162	164.8 M
Quake	146.9 M	16.5 M	13.0 M	189,621	4,076	514	37,279	37,014	9,255	167.3 M

SOAPdenovo assemblies of *Megachile rotundata *124 bp paired end reads. We trimmed the reads before correcting with SOAPdenovo, which greatly improved its performance on our experiments with simulated data. The 'Trimmed only' column includes reads trimmed before and during SOAPdenovo correction. Quake trims reads automatically during correction. Correcting the reads reduces the number of contigs and scaffolds, increases the contig sizes, and allows the assembler to include more reads. Quake corrects more reads than SOAPdenovo which results in a slightly better assembly.

### SNP detection

A second application of short reads that benefits from error correction is detection of variations, such as single nucleotide polymorphisms (SNPs). In such experiments, the genome from which the reads are sequenced differs from a reference genome to which the reads are compared. The first step is to align the reads to the reference genome using specialized methods [8] that will only allow a few mismatches between the read and reference, such as up to two mismatches in a recent study [38]. A read containing a SNP will start with one mismatch already, and any additional differences from the reference due to sequencing errors will make alignment difficult (see Figure 1). Furthermore, the distribution of SNPs in a genome is not uniform and clusters of SNPs tend to appear [39]. Reads from such regions may contain multiple SNPs. If these reads contain any sequencing errors, they will not align causing the highly polymorphic region to be overlooked.

To explore the benefit that error correction with Quake may have on SNP detection, we randomly sampled reads representing 35× from the *E. coli *K12 reads used above. To call SNPs, we aligned the reads to a related reference genome (*E. coli 536 *[GenBank: NC_008253]) with Bowtie [40] using two different modes. We first mapped reads allowing up to two mismatches to resemble the SNP calling pipeline in a recent, large study [38]. We also mapped reads using Bowtie's default mode, which allows mismatches between the reference and read until the sum of the quality values at those mismatches exceeds 70 [40]. We called SNPs using the SAMtools pileup program [41], requiring a Phred-style base call quality ≥40 and a coverage of ≥3 aligned reads. Having a reliable reference genome for both strains of *E. coli *allowed us to compare the SNPs detected using the reads to SNPs detected by performing a whole genome alignment. To call SNPs using the reference genomes, we used the MUMmer utility *dnadiff *which aligns the genomes with MUMmer, identifies the optimal alignment for each region, and enumerates SNPs in aligning regions [37]. We treat these SNPs as the gold standard (though there may be some false positives in improperly aligned regions) in order to compute recall and precision statistics for the read-based SNP calls.

In the first experiment, 128 K additional reads of 4.12 M aligned after correcting with Quake, of which 110 K (85.8%) aligned to SNPs, demonstrating the major benefit of error correction before SNP calling. As seen in Table 5 with these reads mapped, we discovered more SNPs and recall increased at the same level of precision. Supporting the hypothesis that many of these newly discovered SNPs would exist in SNP-dense regions, we found that 62% of the new SNPs were within 10 bp of another SNP, compared to 38% for the entire set of SNPs. On the uncorrected reads, Bowtie's quality-aware alignment policy mapped 165 K (4.9%) more reads than a two mismatch policy. Similarly, many of these new alignments contained SNPs, which led to more SNPs discovered, increasing recall with only a slight drop in precision. Using the quality-aware policy, slightly fewer reads mapped to the reference after error correction because some reads that could not be corrected and were removed could still be aligned. However, 33.7 K new read alignments of corrected reads were found, which allowed the discovery of 518 additional SNPs at the same level of precision. Thus, error correction of the reads using Quake leads to the discovery of more true SNPs using two different alignment policies.

**Table 5:** E. coli SNP calling

Method	Reads mapped	SNPs	Recall	Precision
Two mismatch uncorrected	3.39 M	79,748	0.746	0.987
Two mismatch corrected	3.51 M	80,796	0.755	0.987
Quality-aware uncorrected	3.56 M	85,071	0.793	0.984
Quality-aware corrected	3.55 M	85,589	0.798	0.984

We called SNPs in 35× coverage of 36 bp reads from *E. coli *K12 by aligning the reads to a close relative genome *E. coli 536 *with Bowtie using both a two mismatch and quality-aware alignment policy and calling SNPs with SAMtools pileup. SNPs were validated by comparing the *E. coli *K12 and *E. coli 536 *reference genomes directly. Under both alignment policies, correcting the reads with Quake helps find more true SNPs.

In order to demonstrate the ability of Quake to scale to larger datasets and benefit re-sequencing studies of humans, we corrected 1.7 billion reads from a Korean individual [SRA:SRA008175] [42]. This set includes 1.2 B 36 bp reads and 504 M 75 bp reads. Quake corrected 206 M (11.9%) of these reads, trimmed an additional 75.3 M (4.4%), and removed 344 M (19.9%). Before and after error correction, we aligned the reads to the human genome (NCBI build 37) and called SNPs with Bowtie allowing two mismatches and SAMtools as described above (though requiring the diploid genotype to have quality ≥40 implicitly requires coverage ≥4). Because some putative SNPs had read coverage indicative of a repeat, we filtered out locations with read coverage greater than three times the median coverage of 19, leaving 3,024,283 SNPs based on the uncorrected reads. After error correction, we found 3,083,481 SNPs, an increase of 2.0%. The mean coverage of these SNPs was 20.1 reads, an increase of 4.8% over the coverage of these locations in the alignments of uncorrected reads, which should provide greater accuracy. Thus, Quake helps detect more SNPs in larger diploid genomes as well.

### Data quality

**Figure 2:**
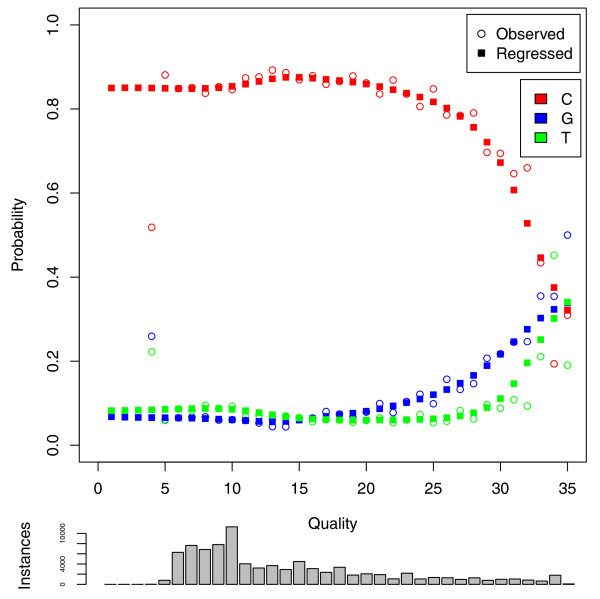
**Adenine error rate**. The observed error rate and predicted error rate after nonparametric regression are plotted for adenine by quality value for a single lane of Illumina sequencing of *Megachile rotundata*. The number of training instances at each quality value are drawn as a histogram below the plot. At low and medium quality values, adenine is far more likely to be miscalled as cytosine than thymine or guanine. However, the distribution at high quality is more uniform.

Our experiences correcting errors in these datasets allowed us to assess the quality of the sequencing data used in a number of interesting ways. First, as has previously been established, nucleotide-specific error rates in Illumina sequencing reads are not uniform [26]. For example, adenines were miscalled far more often as cytosine than thymine or guanine in *Megachile rotundata *(see Figure 2). As exemplified in the figure, error rates also differ significantly by quality value. While miscalls at adenines were highly likely to be cytosines at low quality, errors were closer to uniform at high quality positions in the read. Finally, error rates varied from lane to lane within a sequencing project. For example, the multinomial samples of nucleotide to nucleotide miscall rates for every pair of six lanes from the *Megachile rotundata *sequencing reads differed with unquestionably significant *P*-values using two sample chi square tests.

As sequencing becomes more prevalent in biological research, researchers will want to examine and compare the quality of an instance (single lane, machine run, or whole project) of data generation. Error correction with Quake provides two simple measures of data quality in the number of reads corrected and the number of reads removed. Furthermore, Quake allows the user to search for biases in the data like those described above using bundled analysis scripts on the log of all corrections made. Thus, researchers can detect and characterize problems and biases in their data before downstream analyzes are performed.

## Conclusions

The low cost and high throughput of second-generation sequencing technologies are changing the face of genome research. Despite the many advantages of the new technology, sequencing errors can easily confound analyzes by introducing false polymorphisms and fragmenting genome assemblies. The Quake system detects and corrects sequencing errors by using the redundancy inherent in the sequence data. Our results show that Quake corrects more reads more accurately than previous methods, which in turn leads to more effective downstream analyzes.

One way Quake improves over prior corrections methods is by *q*-mer counting, which uses the quality values assigned to each base as a means of weighting each *k*-mer. The coverage distributions of error and true *k*-mers cannot be separated perfectly according to their number of appearances due to high coverage errors and low coverage genomic regions. Yet, the choice of a cutoff to determine which *k*-mers will be trusted in the correction stage can have a significant affect on downstream applications like genome assembly.

Weighting *k*-mer appearances by quality puts more distance between the two distributions because erroneous *k*-mers generally have lower quality than true *k*-mers. Furthermore, with *q*-mers, the cutoff value separating the two distributions no longer needs to be an integer. For example, at low coverage we might use 0.95 as a cutoff, such that *k*-mers that appear once with high quality bases would be trusted, but those with lower quality would not. Such fine-grained cutoff selection is impossible with simple *k*-mer counting.

Quake includes a sophisticated model of sequencing errors that allows the correction search to examine sets of corrections in order of decreasing likelihood, thus correcting the read more accurately. The model also helps to better identify reads with multiple sets of equally good corrections, which allows the system to avoid mis-correcting and creating a chimeric read. At a minimum, quality values should be included in error correction as a guide to the likely locations of sequencing errors. In each dataset we examined, the rates at which each nucleotide was mis-called to other nucleotides were not uniform and often varied according to quality. Adjusting for these rates provides further improvements in error correction, and distinguishes our method.

We expect Quake will be useful to researchers interested in a number of downstream applications. Correcting reads with Quake improves genome assembly by producing larger and more accurate contigs and scaffolds using the assemblers Velvet [20] and SOAPdenovo [13]. Error correction removes many of the false paths in the assembly graphs caused by errors and helps the assembler to detect overlaps between reads that would have been missed. Eliminating erroneous *k*-mers also significantly reduces the size of the assembly graph, which for large genomes may be the difference between being able to store the graph in a computer's memory or not [13]. In a re-sequencing application, correcting reads with Quake allows Bowtie [40] to align many more reads to locations in the reference genome where there is one or more SNPs. Reads containing variants already have differences from the reference genome; correcting additional differences caused by sequencing errors makes these reads easier to align and then available as input for the SNP calling program. Finally, Quake offers a unique perspective into the quality of the data from a sequencing experiment. The proportion of reads corrected, trimmed, and removed are useful statistics with which experiments can be compared and data quality can be monitored. The output log of corrections can be mined for troubling biases.

On microbial sized genomes, error correction with Quake is fast and unobtrusive for the researcher. On larger datasets, such as a human re-sequencing, it is computationally expensive and requires substantial resources. For the Korean individual reads, we counted *k*-mers on a 20-core computer cluster running Hadoop [43], which required from two to three days. For error correction, the data structure used to store trusted *k*-mers requires 4*^k ^*bits, which is 32 GB for human if *k *= 19. Thus, the correction stage of Quake is best run on a large shared memory machine, where correction is parallelized across multiple threads using OpenMP [44]. Running on 16 cores, this took a few days for the Korean individual dataset. Future work will explore alternative ways to perform this step that would require less memory. This way correction could be parallelized across a larger computer cluster and made more accessible to researchers without a large shared memory machine.

*k*-mer based error correction programs are affected significantly by the cutoff separating true and error *k*-mers. Improvements in *k*-mer classification, such as the *q*-mer counting introduced by Quake, improve the accuracy of error correction. Coverage biases in second-generation sequencing technologies, which are largely inexplicable outside of the affect of local GC content, add to the difficulty [26]. Further characterization of these biases would allow better modeling of *k*-mer coverage and better classification of *k*-mers as true or error. In more repetitive genomes, the probability increases that a *k*-mer that is an artifact of an error actually does occur in the genome. Such *k*-mers are not really misclassified, but may cause Quake to ignore a sequencing error. To improve error correction in these cases, the local context of the *k*-mer in the sequencing reads must be taken into account. Though this was done for Sanger read error correction [15-17], it is not currently computationally and algorithmically feasible for high throughput datasets containing many more reads.

Quake's model for sequencing errors takes into account substantial information about which types of substitution errors are more likely. We considered using Quake to re-estimate the probability of a sequencing error at each quality value before using the quality values for correction. Doing so is difficult because Quake detects many reads that have errors for which it cannot find a valid set of corrections and pinpoint the errors' locations. If Quake re-estimated quality value error probabilities without considering these reads, the error probabilities would be underestimated. Additionally, the benefit of re-estimation is minimal because quality values are mainly used to determine the order in which sets of corrections are considered. Alternatively, passing on more information from the base calling stage, such as the probability that each individual nucleotide is the correct one, would be very helpful. Quake's error model could be made more specific, the need to learn nucleotide specific error rates would be alleviated, and more accurate error correction could be expected.

## Methods

Quake detects and corrects errors in sequencing reads by using *k*-mer coverage to differentiate *k*-mers trusted to be in the genome and *k*-mers that are untrustworthy artifacts of sequencing errors. For reads with untrusted *k*-mers, Quake uses the pattern of trusted and untrusted *k*-mers to localize the errors and searches for the set of corrections with maximum likelihood that make all *k*-mers trusted. The likelihood of a set of corrections to a read is defined by a probabilistic model of sequencing errors incorporating the read's quality values as well as the rates at which nucleotides are miscalled as different nucleotides. Correction proceeds by examining changes to the read in order of decreasing likelihood until a set of changes making all *k*-mers trusted is discovered and found to be sufficiently unambiguous.

### Counting *k*-mers

Counting the number of occurrences of all *k*-mers in the sequencing reads is the first step in the Quake pipeline. *k *must be chosen carefully, but a simple equation suffices to capture the competing goals. Smaller values of *k *provide greater discriminative power for identifying the location of errors in the reads and allow the algorithm to run faster. However, *k *cannot be so small that there is a high probability that one *k*-mer in the genome would be similar to another *k*-mer in the genome after a single nucleotide substitution because these occurrences confound error detection. We recommend setting *k *such that the probability that a randomly selected *k*-mer from the space of $\frac{4^{k}}{2}$ (for odd *k *considering reverse complements as equivalent) possible *k*-mers occurs in a random sequence of nucleotides the size of the sequenced genome *G *is ~0.01. That, is we want *k *such that

(2) $$\frac{2G}{4^{k}}≃0.01$$

which simplifies to

(3) $$k≃\log_{4}200G$$

For an approximately 5 Mbp such as *E. coli*, we set *k *to 15, and for the approximately 3 Gbp human genome, we set *k *to 19 (rounding down for computational reasons). For the human genome, counting all 19-mers in the reads is not a trivial task, requiring >100 GB of RAM to store the *k*-mers and counts, many of which are artifacts of sequencing errors. Instead of executing this computation on a single large memory machine, we harnessed the power of many small memory machines working in parallel on different batches of reads. We execute the analysis using Hadoop [43] to monitor the workflow, and also to sum together the partial counts computed on individual machines using an extension of the MapReduce word counting algorithm [45]. The Hadoop cluster used in these experiments contains 10 nodes, each with a dual core 3.2 gigahertz Intel Xeon processors, 4 GB of RAM, and 367 GB local disk (20 cores, 40 GB RAM, 3.6 TB local disk total).

In order to better differentiate true *k*-mers and error *k*-mers, we incorporate the quality values into *k*-mer counting. The number of appearances of low coverage true *k*-mers and high copy error *k*-mers may be similar, but we expect the error *k*-mers to have lower quality base calls. Rather than increment a *k*-mer's coverage by one for every occurrence, we increment it by the product of the probabilities that the base calls in the *k*-mer are correct as defined by the quality values. We refer to this process as *q-mer counting*. *q*-mer counts approximate the expected coverage of a *k*-mer over the error distribution specified by the read's quality values. By counting *q*-mers, we are able to better differentiate between true *k*-mers that were sequenced to low coverage and error *k*-mers that occurred multiple times due to bias or repetitive sequence.

### Coverage cutoff

**Figure 3:**
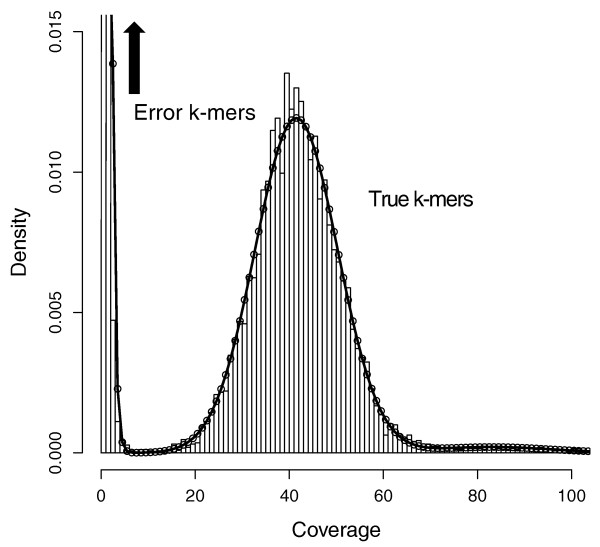
***k*-mer coverage**. 15-mer coverage model fit to 76× coverage of 36 bp reads from *E. coli*. Note that the expected coverage of a *k*-mer in the genome using reads of length *L *will be $\frac{L-k+1}{L}$ times the expected coverage of a single nucleotide because the full *k*-mer must be covered by the read. Above, *q*-mer counts are binned at integers in the histogram. The error *k*-mer distribution rises outside the displayed region to 0.032 at coverage two and 0.691 at coverage one. The mixture parameter for the prior probability that a *k*-mer's coverage is from the error distribution is 0.73. The mean and variance for true *k*-mers are 41 and 77 suggesting that a coverage bias exists as the variance is almost twice the theoretical 41 suggested by the Poisson distribution. The likelihood ratio of error to true *k*-mer is one at a coverage of seven, but we may choose a smaller cutoff for some applications.

A histogram of *q*-mer counts shows a mixture of two distributions - the coverage of true *k*-mers, and the coverage of error *k*-mers (see Figure 3). Inevitably, these distributions will mix and the cutoff at which true and error *k*-mers are differentiated must be chosen carefully [46]. By defining these two distributions, we can calculate the ratio of likelihoods that a *k*-mer at a given coverage came from one distribution or the other. Then the cutoff can be set to correspond to a likelihood ratio that suits the application of the sequencing. For instance, mistaking low coverage *k*-mers for errors will remove true sequence, fragmenting a *de novo *genome assembly and potentially creating mis-assemblies at repeats. To avoid this, we can set the cutoff to a point where the ratio of error *k*-mers to true *k*-mers is high, for example 1,000:1.

In theory, the true *k*-mer coverage distribution should be Poisson, but Illumina sequencing has biases that add variance [26]. Instead, we model true *k*-mer coverage as Gaussian to allow a free parameter for the variance. *k*-mers that occur multiple times in the genome due to repetitive sequence and duplications also complicate the distribution. We found that *k*-mer copy number in various genomes has a 'heavy tail' (meaning the tail of the distribution is not exponentially bounded) that is approximated well by the Zeta distribution [47], which has a single shape parameter. Our full model for true *k*-mer coverage is to sample a copy number from a Zeta distribution, and then sample a coverage from a Gaussian distribution with mean and variance proportional to the chosen copy number.

The error *k*-mer coverage distribution has been previously modeled as Poisson [10]. In data we examined, this distribution also has a heavy tail, which could plausibly be explained if certain sequence motifs were more prone to errors than others due to sequence composition or other variables of the sequencing process. Additionally, by counting *q*-mers, we have real values rather than the integers that Poisson models. We examined a few options and chose the Gamma distribution with free shape and scale parameters to model error *q*-mer counts.

Finally, we include a mixture parameter to determine which of the two distributions a *k*-mer coverage will be sampled from. We fit the parameters of this mixture model by maximizing the likelihood function over the *q*-mer counts using the BFGS algorithm, implemented as the *optim *function in the statistical language R [48]. Figure 3 shows an example fit to 76× coverage of *E. coli*. Using the optimized model, we compute the likelihood ratio of error *k*-mer to true *k*-mer at various coverages and set the cutoff to correspond to the appropriate ratio.

### Localizing errors

**Figure 4:**
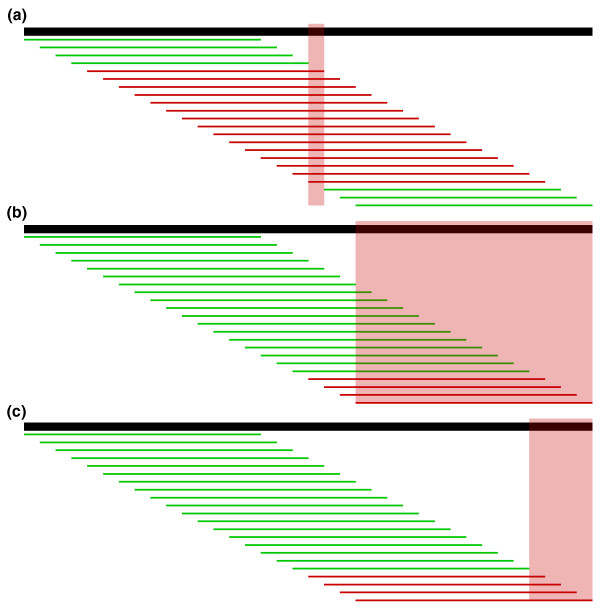
**Localize errors**. Trusted (green) and untrusted (red) 15-mers are drawn against a 36 bp read. In (a), the intersection of the untrusted *k*-mers localizes the sequencing error to the highlighted column. In (b), the untrusted *k*-mers reach the edge of the read, so we must consider the bases at the edge in addition to the intersection of the untrusted *k*-mers. However, in most cases, we can further localize the error by considering all bases covered by the right-most trusted *k*-mer to be correct and removing them from the error region as shown in (c).

Once a cutoff to separate trusted and untrusted *k*-mers has been chosen, all reads containing an untrusted *k*-mer become candidates for correction. In most cases the pattern of untrusted *k*-mers will localize the sequencing error to a small region. For example, in Figure 4a, a single base substitution causes 15 adjacent untrusted 15-mers. To find the most likely region for the sequencing error(s), we take the intersection of a read's untrusted *k*-mers. This method is robust to a few misclassified error *k*-mers, but not to true *k*-mers with low coverage that are classified as untrusted. Thus, if the intersection of the untrusted *k*-mers is empty (which also occurs when there are multiple nearby errors) or a valid correction cannot be found, we try again localizing to the union of all untrusted *k*-mers.

A few more complications are worth noting. If the untrusted *k*-mers reach the edge of the read, there may be more sequencing errors at the edge, so we must extend the region to the edge, as in Figure 4b. In this case and in the case of multiple nearby sequencing errors, we may also benefit from considering every base covered by the right-most trusted *k*-mer and left-most trusted *k*-mer to be correct, and trimming the region as in Figure 4c. Because this heuristic is sensitive to misclassified *k*-mers, we first try to correct in the region shown in Figure 4c, but if no valid set of corrections is found, we try again with the larger region in Figure 4b. Finally, in longer reads we often see clusters of untrusted *k*-mers that do not overlap. We perform this localizing procedure and correction on each of these clusters separately. Altogether, these heuristics for localizing the error in a read vastly decrease the runtime of the algorithm compared to considering corrections across the entire read.

### Sequencing error probability model

After finding a region of the read to focus our correction efforts on, we want to search for the maximum likelihood set of corrections that makes all *k*-mers overlapping the region trusted. First, we must define the likelihood of a set of corrections. Let *O *= *O*_1, _*O*_2_,..., *O_N _*represent the observed nucleotides of the read, and *A *= *A*_1_, *A*_2_,..., *A_N _*the actual nucleotides of the sequenced fragment of DNA. Given the observed nucleotides we would like to evaluate the conditional probability of a potential assignment to *A*. Assuming independence of sequencing errors at nucleotide positions in the read and using Bayes theorem, we can write

(4) $$P(A=a|O=o)=\prod_{i=1}^{N}\frac{P(O_{i}=o_{i}|A_{i}=a_{i})P(A_{i}=a_{i})}{P(O_{i}=o_{i})}$$

Because we compare likelihoods for a single observed read *O *at a time, *P*(*O_i _*= *o_i_*) is the same for all assignments to *A *and is ignored. *P*(*A_i _*= *a_i_*) is defined by the GC% of the genome, which we estimate by counting Gs and Cs in the sequencing reads. Let $p_{i}=1-10^{-\frac{q_{i}}{10}}$ be the probability that the nucleotide at position *i *is accurate, where *q_i _*is the corresponding quality value. Also, let *Eq*(*x*, *y*) be the probability that the base call *y *is made for the nucleotide *x *at quality value *q *given that there has been a sequencing error. Then *P*(*O_i _*= *o_i_|A_i _*= *a_i_*) can be specified as

(5) $$P(O_{i}=o_{i}|A_{i}=a_{i})=\{\begin{array}{ll} p_{i} & \text{if}\;o_{i}=a_{i}\\ \left(1-p_{i})E_{q_{i}}(a_{i},o_{i}\right) & \text{otherwise} \end{array}$$

Modeling sequencing errors with *E *allows for biases in base substitution that are known to exist for the Illumina platform. For example, one study found A to C was the most frequent error, likely because A and C are detected by one laser while G and T are detected by another [26]. Making the substitution distribution conditional upon the quality value allows this substitution bias to vary at different qualities, which was found to occur for Sanger sequencing [49] and here for Illumina (see Figure 2). Although some work has modeled error distributions conditionally on the position of the nucleotide in the read [50], we assume that quality values capture this sequencing cycle effect. Recent base-calling algorithms incorporate this effect on fluorescence intensity measurements explicitly in some way and generate quality values that satisfy our assumption [27-31].

The error matrices *E *are estimated from the sequencing reads as follows. First we initially set $E_{q}(x,y)=\frac{1}{3}\;\;\;\forall q,x,y$ and run the algorithm, counting the corrections by quality value and nucleotide to nucleotide type. During this initial pass, we only make simple, unambiguous corrections by abandoning low quality reads more aggressively and using a greater ambiguity threshold (described below). In order to reduce the variance of our estimate of *E*, we perform kernel smoothing across the quality *q *using a Gaussian kernel [51] with standard deviation two. Let *C_q_*(*x*, *y*) be the number of times actual nucleotide *x *was observed as error nucleotide *y *at quality value *q*, *C_q_*(*x*) be the number of times actual nucleotide *x *was observed as an error at quality value *q*, and *N*(*q*; *u*, *s*) be the probability of *q *from a Gaussian distribution with mean *u *and standard deviation *s*. Then *E *is defined by

(6) $$E_{q}(x,y)=\frac{\sum_{i}C_{q_{i}}(x,y)N(q_{i};q,2)}{\sum_{i}C_{q_{i}}(x)N(q_{i};q,2)}$$

### Correction search

Once we can assign a likelihood to a set of corrections and localize the error(s) to a specific region of the read, we must search for the set with maximum likelihood such that all *k*-mers in the corrected read are trusted. We refer to a set of corrections as *valid *if all resulting *k*-mers are trusted. In order to limit the search space, we consider only sets of corrections for which the ratio of the likelihood of the corrected read to the original is above a fixed threshold (default 10^-6^).

**Figure 5:**
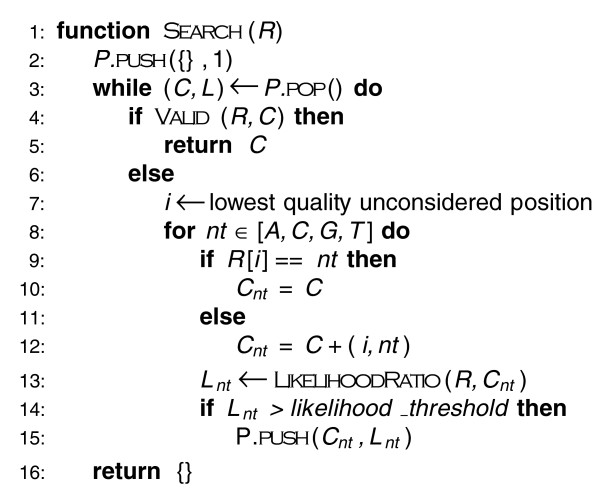
**Correction search algorithm**. Pseudocode for the algorithm to search for the most likely set of corrections that makes all k-mers in the read trusted. *P *is a heap-based priority queue that sorts sets of corrections *C *by their likelihood ratio *L*. The algorithm examines sets of corrections in decreasing order of their likelihood until a set is found that converts all *k*-mers in the read to trusted *k*-mers.

**Figure 6:**
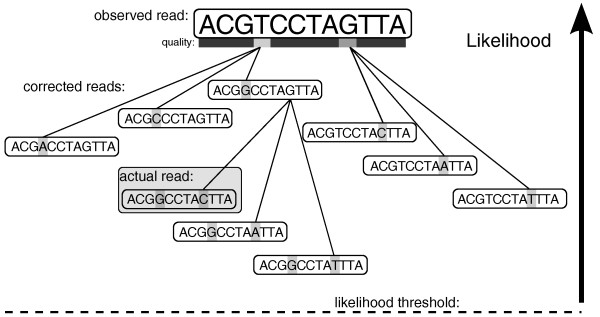
**Correction search**. The search for the proper set of corrections that change an observed read with errors into the actual sequence from the genome can be viewed as exploring a tree. Nodes in the tree represent possible corrected reads (and implicitly sets of corrections to the observed read). Branches in the tree represent corrections. Each node can be assigned a likelihood by our model for sequencing errors as described in the text. Quake's algorithm visits the nodes in order of decreasing likelihood until a valid read is found or the threshold is passed.

Figure 5 outlines the algorithm. To consider sets of corrections in order of decreasing likelihood, the algorithm maintains a heap-based priority queue *P *where each element contains a set of corrections *C *and the ratio of their likelihood to the original read's likelihood *L*. In each iteration through the main loop, the algorithm pops the maximum likelihood set of corrections *C *from the queue *P*. If *C *makes all *k*-mers in the region trusted, then it returns *C*. Otherwise, it examines the next lowest quality read position that has not yet been considered, which we track with minor additional bookkeeping. For each nucleotide substitution at this position, we compute a new likelihood and add the updated set of corrections to the priority queue if its likelihood ratio is above the threshold. If the queue empties without finding a valid set of corrections, we abandon the read. This procedure could alternatively be viewed as searching a tree where nodes are corrected reads and branches represent corrections (see Figure 6).

In practice, we make a few adjustments to this procedure. Reads from repeats may have multiple sets of valid corrections separated by a small likelihood difference so that the true correction is ambiguous. Therefore, we actually continue past the point of finding a valid set of corrections to ensure that another valid set does not exist within a certain likelihood threshold (default 0.1). As described, the algorithm will devote a large majority of its computation effort to the lowest quality reads, which have many potential sets of corrections to consider. In order to balance correction sensitivity with speed, we pre-screen the error region and immediately abandon a read if its error region is filled with low quality base calls. More specifically, in our experiments we found that regions containing ≥13 positions with a probability of error >1% were difficult or impossible to correct quickly, and these reads are abandoned without further effort. For regions containing ≥9 such positions, we increase the likelihood ratio threshold to 10^-3 ^so that we only consider a limited number of corrections before giving up.

In order to run Quake on very large datasets (for example, containing billions of reads), we must be able to determine very quickly whether a set of corrections makes all *k*-mers trusted. We accomplish this by mapping all 4*^k ^k*-mers to an index in a bit array that is set to one if the *k*-mer is trusted and zero otherwise. For 15-mers this bit array uses just 128 MB of space, while it requires 32 GB for 19-mers, which are needed for larger genomes. If memory usage must be reduced, a Bloom filter could be used to hash the trusted *k*-mers in <4 GB at the expense of occasional false positive queries [12].

## Abbreviations

bp: base pair; Gbp: gigabases; Mbp: megabases; SNP: single nucleotide polymorphism.

## Competing interests

The authors declare that they have no competing interests.

## Authors' contributions

DRK conceived and implemented the method and carried out the experiments. MCS assisted with Hadoop. MCS and SLS provided helpful discussion at all stages. DRK, MCS, and SLS wrote the manuscript.
